# Beyond COVID‐19: Equitable epidemiology for studying the impact of maternal infections on neonatal mortality and morbidity

**DOI:** 10.1111/ppe.12892

**Published:** 2022-06-29

**Authors:** Stephanie Jones, Marta Coelho Nunes

**Affiliations:** ^1^ South African Medical Research Council, Vaccines and Infectious Diseases Analytics Research Unit, Faculty of Health Sciences University of the Witwatersrand Johannesburg South Africa; ^2^ Department of Science and Technology/National Research Foundation South African Research Chair Initiative in Vaccine Preventable Diseases, Faculty of Health Sciences University of the Witwatersrand Johannesburg South Africa

There is a special hope and joy surrounding a birth and, therefore, it is especially poignant that neonatal mortality (deaths in the first 28 days of life) and long‐term complications from diseases that occur during the neonatal period remain high throughout the world.^1^ The first month of life is undoubtedly the most vulnerable period during childhood—almost one‐third of all deaths happen on the day of birth and about three‐quarters of neonatal deaths occur during the first week of life.^2^ During the 21st century, we have witnessed incredible development in global health initiatives leading to substantial improvements in health outcomes, especially in child survival. Although the number of overall child deaths is rapidly declining, from 1990 to 2020 the global annual reduction of deaths in children aged 1–59 months was 3.1%, while during the neonatal period, the reduction was 2.6%. As a result, the relative proportion of neonatal deaths among all under‐5 deaths has actually increased from 40% in 1990 to 47% in 2020.^1^ Globally in 2020, approximately 2.4 million newborns died at an estimated average rate of 17 deaths per 1000 live births; in comparison, the estimated probability of dying from 1 to 11 months of age was 11 deaths per 1000 live births, and from 1 year to 4 years of age was 9 deaths per 1000 live births.^1^ To further decrease the burden of neonatal mortality, we will need to address the main causes of these deaths, which are different from the causes in older children.

Neonates primarily die because of preterm birth and intrapartum‐associated complications and infections, including sepsis, meningitis, and pneumonia.^2^ Of the more than half a million annual newborn deaths due to infections, the large majority occur in low‐ and middle‐income countries (LMICs), mostly in sub‐Saharan Africa and south Asia.^1^ Mortality, however, is only a small part of the overall disease burden, as every year there are approximately 7 million episodes of severe infections in newborns, of which an estimated 2.6 million are in sub‐Saharan Africa and 3.5 million in south Asia, often requiring prolonged hospitalisations and leading to long‐term health and neurodevelopmental deficits.^2^ The health of the mother during pregnancy is inextricably linked to neonatal outcomes. Infectious diseases in pregnancy can affect the birth outcome and the newborn health, and are not only directly linked to a high percentage of neonatal mortality, but might also have an underlying role in the aetiology of stillbirth, preterm birth and intra‐partum asphyxia.^1^

A major viral infection in the African continent is human immunodeficiency virus (HIV). Globally, there were an estimated 37.7 million people living with HIV at the end of 2020, over two‐thirds of whom were in Africa.^3^ Many pregnant women living with HIV only start receiving lifelong antiretroviral therapy (ART) when attending antenatal care. While this life‐saving therapy remarkably improved the quality of life of people living with HIV, it means that many newborns are exposed to ART in utero, and the potential risks and benefits of this maternal treatment on birth outcomes and longer‐term offspring health are still unclear. Three articles^4^, ^5^, ^6^ in this special issue of *Paediatric and Perinatal Epidemiology* illustrate the importance of prospective studies from LMICs on infection during pregnancy, and on the impact of new strategies to improve birth outcomes and neonatal health.

Malaba and colleagues^4^ quantify the association between maternal HIV infection status, ART initiation timing, and adverse birth outcomes among a cohort of South African women. Using a bias‐corrected gestational age estimate, they found that women living with HIV compared with those without HIV had similar risks of overall pregnancy loss and of preterm delivery; the risk of delivering small for gestational age births was, however, higher among women living with HIV. Furthermore, among women living with HIV, outcomes were similar by ART initiation timing. The results from this study are reassuring that the increasingly safe and efficacious ART regimens not only lead to improved immunologic status, but also have a less negative impact on pregnancy outcomes compared with earlier treatments.^7^

Nonetheless, the number of infants born HIV‐exposed uninfected is growing worldwide, and these infants have been shown to be at increased risk of mortality, hospitalisation, severe respiratory infections, and potentially longer‐term health outcomes. The rising incidence of childhood obesity is a challenge globally. Maternal obesity is known to influence infant weight gain; however, this association is less clear in children born to women living with HIV, where factors such as in utero exposure to the HIV viral particles and ART may play a role in weight gain.^8^ In this special issue, Bengtson and colleagues^5^ reported that 20% of infants were overweight or obese by 12 months of age in their study in South Africa, and identified maternal body mass index as being positively associated with higher infant weight regardless of maternal HIV status. Therefore, it will be important to include nutritional advice and weight management in routine antenatal and infant care services.

Among neonates and young infants, group B Streptococcus (GBS) is a leading cause of invasive bacterial disease, with a global incidence of 0.49 cases per 1000 live births and an associated case fatality rate of 8.4%; GBS infection may also result in stillbirth and major longer‐term neurodevelopmental sequelae.^9^ Although maternal GBS carriage rates vary substantially by world region, approximately 18% of pregnant women are colonised with GBS in their genitourinary tract that can be vertically transmitted to the newborn. In high‐income countries, pregnant subjects are screened for GBS carriage in the weeks prior to delivery to identify those who could benefit from intrapartum antibiotic prophylaxis. In the absence of such preventive strategies, approximately 1–2% of newborns from mothers colonised with GBS will develop invasive disease.^9^ Despite global advocacy to highlight the GBS burden, research gaps remain and implementation of preventive strategies in LMICs is urgently needed. In another article in this special issue, by evaluating whether diagnosis of maternal GBS colonisation could be done by real‐time polymerase chain reaction (PCR) at the time of delivery, Kugelman and colleagues^6^ concluded that PCR had good sensitivity and specificity compared to culture to detect GBS. They suggested that the need for maternal prophylactic antibiotics could be substantially reduced by using this strategy compared with antepartum universal culture screening or intrapartum risk‐factor assessment. While PCR testing is still expensive and unavailable in many LMIC settings, the cost‐effectiveness of this approach and its future potential use as point‐of‐care should be considered.

To improve maternal health and neonatal survival, it is crucial to ensure that every pregnant woman has access to antenatal care and lifesaving interventions during the intrapartum, delivery, and postnatal periods. These can only be introduced if high‐quality evidence is available to support their implementation and will thus require observational studies and clinical trials in pregnant women and their newborns. Although there is a need to include pregnant women in more studies, because of their inherent vulnerability and perhaps their own reluctance, these studies are often lacking. Studies during pregnancy in LMICs offer not only particular challenges, but also opportunities. Many of the challenges of conducting studies are related to the difficulty in accessing health care. Access and provision of antenatal care in LMICs has its own challenges, including, among others, specific barriers women encounter, limited resources, and inconsistent quality of care. It is estimated that, globally, only 64% of pregnant women attend the previously World Health Organization (WHO) recommended minimum of four antenatal care visits, and in the regions with the highest rates of neonatal and maternal mortality, such as sub‐Saharan Africa (13%) and south Asia (49%), even fewer women received at least four antenatal visits.^10^ The 2016 WHO guidelines re‐emphasise the importance of timely care and recommend doubling the number of antenatal care contacts to eight to reduce perinatal mortality and improve women's care, which will probably lead to even higher disparity in coverage between world regions. Women in LMICs often attend their first antenatal visit in the second trimester which limits the access to early ultrasound examinations and makes accurate gestational age determination difficult. Malaba et al.^4^ highlighted this problem and used quantitative bias analysis to adjust for gestational age misclassification when estimated by last menstrual period or symphysis fundal height.

Several guidelines have been developed for the conduct of studies, reporting of results and use of standardised definitions, such as the ones from the Global Alignment of Immunization Safety Assessment in Pregnancy (GAIA) network specific for safety data in clinical trials of vaccines in pregnant women.^11^ The applicability and success of these are dependent, however, on the quality of record keeping, which needs to be strengthened in LMICs.

Given the well‐known impact of infections in pregnancy, much focus and urgency has been given to studying and mitigating the impact of severe acute respiratory syndrome coronavirus 2 (SARS‐CoV‐2) infection during pregnancy in the last 2 years. Hopefully, this renewed focus on the impact of infectious diseases on pregnancy, birth outcomes, and neonatal growth and development will also extend to other infections that are the cause of many potentially preventable diseases, not just in the neonatal period, but beyond.

## About the authors

**Stephanie Jones** is a medical doctor and is currently a PhD fellow at the Vaccine Preventable Diseases at the Vaccines & Infectious Diseases Analytics Research Unit, Johannesburg, South Africa.

**Marta Coelho Nunes** is an associate professor at the University of the Witwatersrand and the chair of the South African Research Chair on Vaccine Preventable Diseases at the Vaccines & Infectious Diseases Analytics Research Unit, Johannesburg, South Africa.

## AUTHOR CONTRIBUTIONS

Both authors draft and approved the manuscript.
